# Some symmetric identities for the generalized Bernoulli, Euler and Genocchi polynomials associated with Hermite polynomials

**DOI:** 10.1186/s40064-016-3585-3

**Published:** 2016-11-04

**Authors:** Waseem A. Khan, Hiba Haroon

**Affiliations:** Department of Mathematics, Integral University, Lucknow, 226026 India

**Keywords:** Hermite polynomials, Bernoulli polynomials, Euler polynomials and Genocchi polynomials, Hermite–Bernoulli polynomials, Hermite–Euler polynomials, Hermite–Genocchi polynomials, Symmetric identities, 05A15, 11B68, 33C45, 33C99

## Abstract

In 2008, Liu and Wang established various symmetric identities for Bernoulli, Euler and Genocchi polynomials. In this paper, we extend these identities in a unified and generalized form to families of Hermite–Bernoulli, Euler and Genocchi polynomials. The procedure followed is that of generating functions. Some relevant connections of the general theory developed here with the results obtained earlier by Pathan and Khan are also pointed out.

## Background

Let $H_n(x,y)$ be denoted by the 2-variable Kampé de Fériet generalization of the Hermite polynomials Bell (1934) and Dattoli et al. (1999) defined as

1 $$\begin{aligned} H_n(x,y)=n!\sum \limits _{r=0}^{[\frac{n}{2}]}\frac{y^rx^{n-2r}}{r!(n-2r)!} \end{aligned}$$

These polynomials are usually defined by the generating function as

2 $$\begin{aligned} e^{xt+yt^2}=\sum \limits _{n=0}^{\infty }H_n(x,y)\frac{t^n}{n!} \end{aligned}$$

and reduce to the ordinary Hermite polynomials $H_n(x)$ (see Andrews 1985) when $y=-1$ and *x* is replaced by 2*x*.

The generalized Bernoulli polynomials $B_{n}^{(\alpha )}(x)$ of order $\alpha$, $\alpha \in \mathbb C$, the generalized Euler polynomials $E_n^{(\alpha )}(x)$ of order $\alpha$, $\alpha \in \mathbb C$ and the generalized Genocchi polynomials $G_n^{(\alpha )}(x)$ of order $\alpha$, $\alpha \in \mathbb C$, each of degree *n* as well as in $\alpha$ are defined respectively by the following generating functions (see Dere and Simsek 2015, [Erdelyi et al. (1953), vol. 3, p. 253 et seq.], [Luke (1969), Section 2.8] and Luo et al. 2003; Pathan 2012; Pathan and Khan 2014, 2015, 2016; Simsek 2010; Srivastava et al. 2012):

3 $$\begin{aligned} \left( \frac{t}{e^t-1}\right) ^{\alpha }e^{xt}=\sum \limits _{n=0}^{\infty }B_{n}^{(\alpha )}(x)\frac{t^n}{n!},\quad (|t| < 2\pi ; 1^{\alpha }=1) \end{aligned}$$

4 $$\begin{aligned} \left( \frac{2}{e^t+1}\right) ^{\alpha }e^{xt}=\sum \limits _{n=0}^{\infty }E_{n}^{(\alpha )}(x)\frac{t^n}{n!},\quad (|t| < \pi ; 1^{\alpha }=1) \end{aligned}$$

and

5 $$\begin{aligned} \left( \frac{2t}{e^t+1}\right) ^{\alpha }e^{xt}=\sum \limits _{n=0}^{\infty }G_{n}^{(\alpha )}(x)\frac{t^n}{n!}, \quad (|t| < \pi ; 1^{\alpha }=1) \end{aligned}$$

It is easy to see that

$$\begin{aligned} B_n^{(\alpha )}(x)=B_{n}^{(\alpha )}(x;1), \quad E_n^{(\alpha )}(x)=E_{n}^{(\alpha )}(x;1) \end{aligned}$$

and

6 $$\begin{aligned} G_n^{(\alpha )}(x)=G_{n}^{(\alpha )}(x;1)\quad(n\in \mathbb N) \end{aligned}$$

Moreover

7 $$\begin{aligned} B_n (1)-B_n=\delta _{(n,1)},\quad n\ge 0 \end{aligned}$$

For each $k\in \mathbb N_0$, $S_k(n)$ defined by

8 $$\begin{aligned} S_k(n)=\sum \limits _{j=0}^{n}j^k \end{aligned}$$

is called the sum of integer powers Tuenter (2001). The exponential generating function for $S_k(n)$ is given by

9 $$\begin{aligned} \sum \limits _{k=0}^{\infty }S_k(n)\frac{t^k}{k!}=\frac{e^{(n+1)t}-1}{e^t-1} \end{aligned}$$

For each $$k\in \mathbb N_0$$, $$ T_k(n)$$ defined by

10 $$\begin{aligned} T_k(n)=\sum \limits _{j=0}^{n}(-1)^j {j^k} \end{aligned}$$

is called the alternating sum. The exponential generating function for $T_k(n)$ is

11 $$\begin{aligned} \sum \limits _{k=0}^{\infty }T_k(n)\frac{t^k}{k!}=\frac{1-{(-e^t)}^{(n+1)}}{1+e^t} \end{aligned}$$

The following are some special values

12 $$\begin{aligned}&S_k (0)=T_k(0)=\delta _{(0,k)}\nonumber \\&S_k(1)=0^k+1^k=\delta _{(0,k)}+1, \quad  T_k (1)=0^k-1^k=\delta _{(0,k)}-1 \end{aligned}$$

where $\delta _{(i,j)}$ is the Kronecker delta defined by $\delta _{(i,j)}=1$ for $i=j$ and $\delta _{(i,j)}=0$ for $i\ne j$.

A close relation of the power sum and the Bernoulli polynomials, also the alternate sum and the Euler polynomials, can be seen in Abramowitz and Stegun [1972, Eq.(23.1.4)] as follows

13 $$\begin{aligned} S_k(n)=\sum \limits _{i=0}^{n}{i^k}=\frac{{B_{k+1}(n+1)}-{B_{k+1}}}{k+1} \end{aligned}$$

14 $$\begin{aligned} (-1)^nT_k(n)=\sum \limits _{i=0}^{n}(-1)^{n-i}i^k=\frac{E_k{(n+1)}+(-1)^nE_k{(0)}}{2} \end{aligned}$$

where *n* and *k* are nonnegative integers.

The hyperbolic cotangent and the hyperbolic tangent (Weisstein http://mathworld.wolfram.com) respectively are defined by

15 $$\begin{aligned} cothz=\frac{e^{2z}+1}{e^{2z}-1}=\sum \limits _{n=-1}^{\infty }\frac{2^n(B_{n+1}+B_{n+1}(1))}{(n+1)!}z^n=\sum \limits _{n=0}^{\infty }\frac{2^{n-1}(B_n+B_n(1))}{n!}z^{n-1} \end{aligned}$$

and

16 $$\begin{aligned} tanhz=\frac{e^{2z}-1}{e^{2z}+1}=\sum \limits _{n=1}^{\infty }\frac{2^{n+1}(2^{n+1}-1)B_{n+1}}{(n+1)!}z^n \end{aligned}$$

Therefore

17 $$\begin{aligned} tanhz=-\sum \limits _{n=1}^{\infty }E_n (0)\frac{{(2z)}^n}{n!} \end{aligned}$$

where

$$\begin{aligned} E_n(0)=\frac{2(1-2^{n+1})}{(n+1)}B_{n+1}, ~n\ge 0, \quad ({\text{see}}\,[1,\,{\text{Eq.}}\,(23.1.20)]).\end{aligned}$$

Pathan and Khan (2015) introduced the generalized Hermite–Bernoulli polynomials for two variables ${}_HB_n^{(\alpha )}(x,y)$ defined by

18 $$\begin{aligned} \left( \frac{t}{e^t-1}\right) ^{\alpha }e^{xt+yt^2}=\sum \limits _{n=0}^{\infty }{}_HB_n^{(\alpha )}(x,y)\frac{t^n}{n!} \end{aligned}$$

which is essentially a generalization of Bernoulli numbers, Bernoulli polynomials, Hermite polynomials and Hermite–Bernoulli polynomials ${_HB}_n(x,y)$ introduced by Dattoli et al. [1999, p. 386 (1.6)] in the form

19 $$\begin{aligned} \left( \frac{t}{e^t-1}\right) e^{xt+yt^2}=\sum \limits _{n=0}^{\infty }{}_HB_n(x,y)\frac{t^n}{n!} \end{aligned}$$

The purpose of this paper is to give some general symmetry identities for generalized Hermite–Euler, Hermite–Genocchi and mixed type polynomials by using different analytical means on their respective generating functions. These results extend some known identities of Hermite, Bernoulli, Euler, Genocchi and mixed type polynomials studied by Dattoli et al. (1999), Liu and Wang (2009), Pathan (2012) and Pathan and Khan (2014, 2015, 2016).

## Symmetry identities for generalized Hermite–Euler polynomials

In this section, we establish general symmetry identities for the generalized Hermite–Euler polynomials ${}_HE_{n}^{(\alpha )}(x,y)$, of order$\alpha$ and alternate power sum. Throughout this section $\alpha$ will be taken as an arbitrary real or complex parameter.

### **Theorem 1**

*For integers* $n\ge 0$, $a\ge 1$ *and* $b\ge 1,$ if *a* *and* *b* *have* *the same parity, then the following identity holds true:*

20 $$\begin{aligned}&\sum \limits _{k=0}^{n}\left( \begin{array}{lll}n\\ k\end{array}\right) a^{n-k}b^{k}{}_HE_{n-k}^{(\alpha )}(bx,b^{2}z)\sum \limits _{i=0}^{k}\left( \begin{array} {lll}k\\ i\end{array}\right) T_i(a-1)E_{k-i}^{(\alpha -1)}(ay)\nonumber \\&\quad =\sum \limits _{k=0}^{n}\left( \begin{array}{lll}n\\ k\end{array}\right) a^{k}b^{n-k} {}_HE_{n-k}^{(\alpha )}(ax,a^{2}z)\sum \limits _{i=0}^{k}\left( \begin{array}{lll}k\\ i\end{array}\right) T_i(b-1)E_{k-i}^{(\alpha -1)}(by) \end{aligned}$$

### Proof

Let $$G(t)=:\frac{2^{2{\alpha }-1}(1-(-e^{bt})^a)e^{ab(x+y)t+a^2b^2zt^2}}{(e^{at}+1)^{\alpha }(e^{bt}+1)^{\alpha }}$$

$$\begin{aligned}G(t)&={\left( \frac{2}{e^{at}+1}\right) ^{\alpha }}e^{abxt+a^2b^2zt^2}{\left( \frac{1-{(-e^{bt})}^a}{1+e^{bt}}\right) }{\left( \frac{2}{e^{bt}+1}\right) ^{{\alpha }-1}}e^{abyt} \\& ={\left( \sum \limits _{n=0}^{\infty }{}_HE_{n}^{(\alpha )}(bx,b^{2}z)\frac{(at)^n}{n!}\right) }{\left( \sum \limits _{i=0}^{\infty }T_i{(a-1)}\frac{(bt)^i}{i!}\right) } \left( \sum \limits _{k=0}^{\infty }E_k^{(\alpha -1)}{(ay)\frac{(bt)^k}{k!}}\right) \\&={\left( {\sum \limits _{n=0}^{\infty }{}_HE_{n}^{(\alpha )}(bx,b^{2}z)\frac{(at)^n}{n!}}\right) }{\left( \sum \limits _{k=0}^{\infty }\sum \limits _{i=0}^{\infty } {b^{i+k}}T_i{(a-1)}E_k^{(\alpha -1)}(ay)\frac{t^{i+k}}{i!k!}\right) } \\&={\sum \limits _{n=0}^{\infty }{}_HE_{n}^{(\alpha )}(bx,b^{2}z)\frac{(at)^n}{n!}}{\sum \limits _{k=0}^{\infty }{b^k}\sum \limits _{i=0} ^{k}\left( \begin{array}{lll}k\\ i\end{array}\right) T_i{(a-1)}E_{k-i}^{(\alpha -1)}(ay)\frac{t^k}{k!}} \\ G(t)&={\sum \limits _{n=0}^{\infty }\sum \limits _{k=0}^{\infty }{a^n}{b^k}{}_HE_{n}^{(\alpha )}(bx,b^{2}z)\sum \limits _{i=0}^{k} \left( \begin{array}{lll}k\\ i\end{array}\right) T_i{(a-1)}E_{k-i}^{(\alpha -1)}(ay)\frac{t^{n+k}}{k!n!}} \end{aligned}$$

Replacing *n* by $n-k$ in the R.H.S. of above equation, we get

21 $$\begin{aligned} G(t)={\sum \limits _{n=0}^{\infty }\left[ \sum \limits _{k=0}^{n}\left( \begin{array}{lll}n\\ k\end{array}\right) {a^{n-k}}{b^k}{}_HE_{n-k} ^{(\alpha )}(bx,b^{2}z)\sum \limits _{i=0}^{k}\left( \begin{array}{lll}k\\ i\end{array}\right) T_i{(a-1)}E_{k-i}^{(\alpha -1)}(ay)\right] }\frac{t^{n}}{n!} \end{aligned}$$

Using a similar plan, we get

22 $$\begin{aligned} G(t)={\sum \limits _{n=0}^{\infty }\left[ \sum \limits _{k=0}^{n}\left( \begin{array}{lll}n\\ k\end{array}\right) {b^{n-k}}{a^k}{}_HE_{n-k} ^{(\alpha )}(ax,a^{2}z)\sum \limits _{i=0}^{k}\left( \begin{array}{lll}k\\ i\end{array}\right) T_i{(b-1)}E_{k-i}^{(\alpha -1)}(by)\right] }\frac{t^{n}}{n!} \end{aligned}$$

By comparing the coefficients of $\frac{t^n}{n!}$ in the R.H.S of above Eqs. (21) and (22), we arrive at the desired result.

### *Remark 1*

For $z=0$ in Theorem (1), the result reduces to known result of Liu and Wang [2009, p. 3348, (2.1)].

### **Corollary 1**

*For integers* $n\ge 0$, $a\ge 1$ *and* $b\ge 1$ , *if* *a* *and* *b* *have the same parity, then the following identity holds true:*

23 $$\begin{aligned}&\sum \limits _{k=0}^{n}\left( \begin{array}{lll}n\\ k\end{array}\right) a^{n-k}b^{k}E_{n-k}^{(\alpha )}(bx)\sum \limits _{i=0}^{k}\left( \begin{array} {lll}k\\ i\end{array}\right) T_i(a-1)E_{k-i}^{(\alpha -1)}(ay) \nonumber \\&\quad =\sum \limits _{k=0}^{n}\left( \begin{array}{lll}n\\ k\end{array}\right) a^{k}b^{n-k} E_{n-k}^{(\alpha )}(ax)\sum \limits _{i=0}^{k}\left( \begin{array}{lll}k\\ i\end{array}\right) T_i(b-1)E_{k-i}^{(\alpha -1)}(by) \end{aligned}$$

### **Theorem 2**

*For integers* $n\ge 0$, $a\ge 1$ *and* $b\ge 1$ , *if* *a* *is odd and* *b* *is even, then the following identity holds true:*

24 $$\begin{aligned}&\sum \limits _{k=0}^{n}\left( \begin{array}{lll}n\\ k\end{array}\right) a^{n-k}b^{k}{}_HE_{n-k}^{(\alpha )}(bx,b^{2}z)\sum \limits _{i=0}^{k}\left( \begin{array}{lll}k\\ i\end{array}\right) T_i(a-1)E_{k-i}^{(\alpha -1)}(ay)\nonumber \\&\quad =-2\sum \limits _{l=0,l\ne n}^{n+1}\left( \begin{array}{lll}{n+1}\\ ~l~\end{array}\right) \frac{B_{n+1-l}}{n+1} a^{n-l}\sum \limits _{k=0}^{l} \left( \begin{array}{lll}l\\ k\end{array}\right) b^{n-k}a^{k}{}_HE_{l-k}^{(\alpha )}(ax,a^{2}z)\nonumber \\&\qquad \times \sum \limits _{i=0}^{k}\left( \begin{array}{lll}k\\ i\end{array}\right) T_i(b-1)E_{k-i}^{(\alpha -1)}(by) \end{aligned}$$

### Proof

Let $$G(t)=:\frac{2^{2{\alpha }-1}(1-(-e^{bt})^a)e^{ab(x+y)t+a^2b^2zt^2}}{(e^{at}+1)^{\alpha }(e^{bt}+1)^{\alpha }}$$

$$\begin{aligned}G(t)&={\left( \frac{2}{e^{at}+1}\right) ^{\alpha }}e^{abxt+a^2b^2zt^2}{\left( \frac{1-{(-e^{bt})}^a}{1+e^{bt}}\right) }{\left( \frac{2}{e^{bt}+1}\right) ^{{\alpha }-1}}e^{abyt}\\ & ={\left( \sum \limits _{n=0}^{\infty }{}_HE_{n}^{(\alpha )}(bx,b^{2}z)\frac{(at)^n}{n!}\right) }{\left( \sum \limits _{i=0}^{\infty }T_i{(a-1)}\frac{(bt)^i}{i!}\right) } \left( \sum \limits _{k=0}^{\infty }E_k^{(\alpha -1)}{(ay)\frac{(bt)^k}{k!}}\right) \\& ={\left( \sum \limits _{n=0}^{\infty }{}_HE_{n}^{(\alpha )}(bx,b^{2}z)\frac{(at)^n}{n!}\right) }{\left( \sum \limits _{k=0}^{\infty }{b^k}\sum \limits _{i=0} ^{k}\left( \begin{array}{lll}k\\ i\end{array}\right) T_i{(a-1)}E_{k-i}^{(\alpha -1)}(ay)\frac{t^k}{k!}\right) } \end{aligned}$$

Replacing *n* by $n-k$ in the R.H.S. of above equation, we get

25 $$\begin{aligned} G(t)={\sum \limits _{n=0}^{\infty }\left[ \sum \limits _{k=0}^{n}\left( \begin{array}{lll}n\\ k\end{array}\right) {a^{n-k}}{b^k}{}_HE_{n-k} ^{(\alpha )}(bx,b^{2}z)\sum \limits _{i=0}^{k}\left( \begin{array}{lll}k\\ i\end{array}\right) T_i{(a-1)}E_{k-i}^{(\alpha -1)}(ay)\right] }\frac{t^{n}}{n!} \end{aligned}$$

Since *G*(*t*) is not symmetric in *a* and *b*, thus *G*(*t*) can also be expanded as

26 $$\begin{aligned}G(t)&={\left( \frac{2}{e^{bt}+1}\right) ^{\alpha }}e^{abxt+a^2b^2zt^2}{\left( \frac{1-{(-e^{at})}^b}{1+e^{at}}\right) }{\left( \frac{1-{(-e^{bt})}^a}{1-{(-e^{at})}^b} \right) }{\left( \frac{2}{e^{at}+1}\right) ^{{\alpha }-1}}e^{abyt}\nonumber \\& ={\left( \sum \limits _{l=0}^{\infty }{}_HE_{l}^{(\alpha )}(ax,a^{2}z)\frac{(bt)^l}{l!}\right) }{\left( \sum \limits _{i=0}^{\infty }T_i{(b-1)}\frac{(at)^i}{i!}\right) } \left( -\sum \limits _{n=0}^{\infty }\frac{B_n+B_n(1)}{n!}(abt)^{n-1}\right) \nonumber \\&\qquad \times \left( \sum \limits _{k=0}^{\infty }E_k^{(\alpha -1)}{(by)\frac{(at)^k}{k!}}\right) \nonumber \\G(t)&=\left( -\sum \limits _{n=0}^{\infty }\frac{B_n+B_n(1)}{n!}(abt)^{n-1}\right) {\left( \sum \limits _{l=0}^{\infty }{}_HE_{l}^{(\alpha )}(ax,a^{2}z)\frac{(bt)^l}{l!} \right) }\nonumber \\&\qquad \times {\left( \sum \limits _{k=0}^{\infty }\sum \limits _{i=0}^{k}a^k\left( \begin{array}{lll}k\\ i\end{array}\right) T_i{(b-1)}E_{k-i}^{(\alpha -1)}(by)\frac{t^k}{k!} \right) } \end{aligned}$$

Using identity (7) and comparing the coefficients of $\frac{t^n}{n!}$ in the R.H.S. of Eqs. (25) and (26), we get the desired result.

### *Remark 2*

For $$ z=0$$ in Theorem (2), the result reduces to known result of Liu and Wang [2009, p. 3349, Theorem (4)].

### **Corollary 2**

*For integers* $n\ge 0$, $a\ge 1$ *and* $b\ge 1$ , *if* *a* *is odd and* *b* *is even, then the following identity holds true:*

27 $$\begin{aligned}&\sum \limits _{k=0}^{n}\left( \begin{array}{lll}n\\ k\end{array}\right) a^{n-k}b^{k}E_{n-k}^{(\alpha )}(bx)\sum \limits _{i=0}^{k}\left( \begin{array}{lll}k\\ i\end{array}\right) T_i(a-1)E_{k-i}^{(\alpha -1)}(ay)\nonumber \\&\quad =-2\sum \limits _{l=0,l\ne n}^{n+1}\left( \begin{array}{lll}{n+1}\\ l\end{array}\right) \frac{B_{n+1-l}}{n+1} a^{n-l}\sum \limits _{k=0}^{l} \left( \begin{array}{lll}l\\ k\end{array}\right) b^{n-k}a^{k}E_{l-k}^{(\alpha )}(ax)\nonumber \\&\qquad \times \sum \limits _{i=0}^{k}\left( \begin{array}{lll}k\\ i\end{array}\right) T_i(b-1)E_{k-i}^{(\alpha -1)}(by) \end{aligned}$$

### **Theorem 3**

*For integers* $n\ge 1$ , $a\ge 1$ *and* $b\ge 1$, *if* *a* *is even and* *b* *is odd, then the following identity holds true:*

28 $$\begin{aligned}&\sum \limits _{k=0}^{n}\left( \begin{array}{lll}n\\ k\end{array}\right) a^{n-k} b^k{}_HE_{n-k}^{(\alpha )}(bx,b^2 z)\sum \limits _{i=0}^{k}\left( \begin{array} {lll}k\\ i\end{array}\right) T_i(a-1)E_{k-i}^{(\alpha -1)}(ay)\nonumber \\&\quad =\sum \limits _{l=0}^{n-1}\left( \begin{array}{lll}n\\ l\end{array}\right) E_{n-l}(0)a^{n-l} \sum \limits _{k=0}^{l}\left( \begin{array}{lll}l\\ k\end{array}\right) b^{n-k} a^k {}_HE_{l-k}^{(\alpha )} (ax,a^2 z)\nonumber \\&\qquad \times \sum \limits _{i=0}^k\left( \begin{array}{lll}k\\ i\end{array}\right) T_i (b-1)E_{k-i}^{(\alpha -1)}(by) \end{aligned}$$

### Proof

Let

29 $$\begin{aligned}G(t)&=:\frac{2^{2{\alpha }-1}(1-(-e^{bt})^a)e^{ab(x+y)t+a^2b^2zt^2}}{(e^{at}+1)^{\alpha }(e^{bt}+1)^{\alpha }}\nonumber \\G(t)&={\left( \frac{2}{e^{at}+1}\right) ^{\alpha }}e^{abxt+a^2b^2zt^2}{\left( \frac{1-{(-e^{bt})}^a}{1+e^{bt}}\right) }{\left( \frac{2}{e^{bt}+1}\right) ^{{\alpha }-1}}e^{abyt}\nonumber \\&={\left( \sum \limits _{n=0}^{\infty }{}_HE_{n}^{(\alpha )}(bx,b^{2}z)\frac{(at)^n}{n!}\right) }{\left( \sum \limits _{i=0}^{\infty }T_i{(a-1)}\frac{(bt)^i}{i!}\right) } \left( \sum \limits _{k=0}^{\infty }E_k^{(\alpha -1)}{(ay)\frac{(bt)^k}{k!}}\right) \nonumber \\ \end{aligned}$$

Another expansion of *G*(*t*) is as follows :

30 $$\begin{aligned}G(t)&={\left( \frac{2}{e^{bt}+1}\right) ^{\alpha }}e^{abxt+a^2b^2zt^2}{\left( \frac{1-{(-e^{at})}^b}{1+e^{at}}\right) } {\left( \frac{1-{(-e^{bt})}^a}{1-{(-e^{at})}^b}\right) }{\left( \frac{2}{e^{at}+1}\right) ^{{\alpha }-1}}e^{abyt}\nonumber \\ &=\left( \sum \limits _{l=0}^{\infty }{}_HE_{l}^{(\alpha )}(ax,a^{2}z)\frac{(bt)^l}{l!}\right) \left( -\sum \limits _{n=0}^{\infty } \frac{B_n+B_n(1)}{n!}(abt)^{n-1}\right) \left( \sum \limits _{i=0}^{\infty }T_i{(b-1)}\frac{(at)^i}{i!}\right) \nonumber \\&\quad \times \left( \sum \limits _{k=0}^{\infty }E_k^{(\alpha -1)} {(by)\frac{(at)^k}{k!}}\right) \nonumber \\G(t)&=\left( \sum \limits _{l=0}^{\infty }{}_HE_{l}^{(\alpha )}(ax,a^{2}z)\frac{(bt)^l}{l!}\right) \left( -\sum \limits _{n=0}^{\infty } \frac{B_n+B_n(1)}{n!}(abt)^{n-1}\right) \nonumber \\&\quad \times \sum \limits _{k=0}^{\infty }\sum \limits _{i=0}^{k}\left( \begin{array}{lll}k\\ i\end{array}\right) a^kT_i{(b-1)}E_{k-i}^{(\alpha -1)}(by)\frac{t^k}{k!} \end{aligned}$$

Now using Eq. (17) and identity (7) and comparing the coefficients of $\frac{t^n}{n!}$ in the R.H.S. of Eqs. (29) and (30), we arrive at the desired result.

### *Remark 3*

For $z=0$ in Theorem (3), the result reduces to known result of Liu and Wang [2009, p. 3350, (2.11)].

### **Corollary 3**

*For integers* $n\ge 1,$ $a\ge 1$ *and* $b\ge 1$, *if* *a* *is even and* *b* *is odd, then the following identity holds true:*

31 $$\begin{aligned}&\sum \limits _{k=0}^{n}\left( \begin{array}{lll}n\\ k\end{array}\right) a^{n-k} b^kE_{n-k}^{(\alpha )}(bx)\sum \limits _{i=0}^{k}\left( \begin{array} {lll}k\\ i\end{array}\right) T_i(a-1)E_{k-i}^{(\alpha -1)}(ay)\nonumber \\&\quad =\sum \limits _{l=0}^{n-1}\left( \begin{array}{lll}n\\ l\end{array}\right) E_{n-l}(0)a^{n-l} \sum \limits _{k=0}^{l}\left( \begin{array}{lll}l\\ k\end{array}\right) b^{n-k} a^k E_{l-k}^{(\alpha )}(ax)\nonumber \\&\qquad \times \sum \limits _{i=0}^k\left( \begin{array}{lll}k\\ i\end{array}\right) T_i (b-1)E_{k-i}^{(\alpha -1)}(by) \end{aligned}$$

### **Theorem 4**

*For integers* $n\ge 0$, $a\ge 1$ *and* $b\ge 1$, *if* *a* *and* *b* *have same parity, then the following identity holds true:*

32 $$\begin{aligned}&\sum \limits _{k=0}^{n}\left( \begin{array}{lll}n\\ k\end{array}\right) {a^k}{b^{n-k}}\sum \limits _{i=0}^{a-1}{(-1)^i}{}_HE_k^{(\alpha )}(bx+{\frac{b}{a}}i,b^2z) E_{n-k}^{(\alpha -1)}(ay)\nonumber \\&\quad =\sum \limits _{k=0}^{n}\left( \begin{array}{lll}n\\ k\end{array}\right) {a^{n-k}}{b^k}\sum \limits _{i=0}^{b-1}{(-1)^i}{}_HE_k^{(\alpha )} (ax+{\frac{a}{b}}i,a^2z)E_{n-k}^{(\alpha -1)}(by) \end{aligned}$$

### Proof

Let

$$\begin{aligned} G(t)=:\frac{2^{2{\alpha }-1}(1-(-e^{bt})^a)e^{ab(x+y)t+a^2b^2zt^2}}{(e^{at}+1)^{\alpha }(e^{bt}+1)^{\alpha }} \end{aligned}$$

We expand *G*(*t*) as follows :

$$\begin{aligned}G(t)&={\left( \frac{2}{e^{at}+1}\right) ^{\alpha }}e^{abxt+a^2b^2zt^2}{\left( \frac{1-{(-e^{bt})}^a}{1+e^{bt}}\right) }{\left( \frac{2}{e^{bt}+1}\right) ^{{\alpha }-1}}e^{abyt}\\& ={\left( \frac{2}{e^{at}+1}\right) ^{\alpha }}e^{abxt+a^2b^2zt^2}{\left( \sum \limits _{i=0}^{a-1}(-1)^ie^{bti}\right) }{\left( \sum \limits _{n=0}^{\infty }E_n^ {(\alpha -1)}(ay)\frac{(bt)^n}{n!}\right) }\\&={\left( \sum \limits _{i=0}^{a-1}(-1)^i{\left( \frac{2}{e^{at}+1}\right) }^{\alpha }e^{(bx+\frac{b}{a}i)at+a^2b^2zt^2}\right) }{\left( \sum \limits _{n=0}^{\infty } E_{n}^{(\alpha -1)}(ay)\frac{(bt)^n}{n!}\right) }\\G(t)&={\left( \sum \limits _{i=0}^{a-1}(-1)^i\sum \limits _{k=0}^{\infty }{}_HE_k^{(\alpha )}(bx+\frac{b}{a}i;b^2z)\frac{(at)^k}{k!}\right) }{\left( \sum \limits _{n=0} ^{\infty }E_{n}^{(\alpha -1)}(ay)\frac{(bt)^n}{n!}\right) } \end{aligned}$$

Replacing *n* by $n-k$ in the R.H.S. of above equation, we get

33 $$\begin{aligned} G(t)=\sum \limits _{n=0}^{\infty }\left[ \sum \limits _{k=0}^{n}\left( \begin{array}{lll}n\\ k\end{array}\right) a^kb^{n-k}\sum \limits _{i=0}^ {a-1}(-1)^i{}_HE_k^{(\alpha )}(bx+\frac{b}{a}i;b^2z)E_{n-k}^{(\alpha -1)}(ay)\right] \frac{t^n}{n!} \end{aligned}$$

Using a similar plan, we obtain

34 $$\begin{aligned} G(t)=\sum \limits _{n=0}^{\infty }\left[ \sum \limits _{k=0}^{n}\left( \begin{array}{lll}n\\ k\end{array}\right) b^ka^{n-k}\sum \limits _{i=0}^ {b-1}(-1)^i{}_HE_k^{(\alpha )}(ax+\frac{a}{b}i;a^2z)E_{n-k}^{(\alpha -1)}(by)\right] \frac{t^n}{n!} \end{aligned}$$

By comparing the coefficients of $\frac{t^n}{n!}$ in the R.H.S. of last two Eqs. (33) and (34), we arrive at the desired result.

### *Remark 4*

For $z=0$ in Theorem (4), the result reduces to known result of Liu and Wang [2009, Theorem 2.10].

### **Corollary 4**

*For integers* $n\ge 0$, $a\ge 1$ *and* $b\ge 1$, *if* *a* *and* *b* *have same parity, then the following identity holds true:*

35 $$\begin{aligned}&\sum \limits _{k=0}^{n}\left( \begin{array}{lll}n\\ k\end{array}\right) {a^k}{b^{n-k}}\sum \limits _{i=0}^{a-1}{(-1)^i}E_k^{(\alpha )}(bx+{\frac{b}{a}}i) E_{n-k}^{(\alpha -1)}(ay)\nonumber \\&\quad =\sum \limits _{k=0}^{n}\left( \begin{array}{lll}n\\ k\end{array}\right) {a^{n-k}}{b^k}\sum \limits _{i=0}^{b-1}{(-1)^i}E_k^{(\alpha )} (ax+{\frac{a}{b}}i)E_{n-k}^{(\alpha -1)}(by) \end{aligned}$$

### **Theorem 5**

*For integers* $n\ge 0,$ $a\ge 1$ *and* $b\ge 1$, *if* *a* *is odd and* *b* *is even, then the following identity holds true:*

36 $$\begin{aligned}&\sum \limits _{k=0}^{n}\left( \begin{array}{lll}n\\ k\end{array}\right) {a^k}{b^{n-k}}\sum \limits _{i=0}^{a-1}{(-1)^i}{}_HE_k^{(\alpha )}(bx+{\frac{b}{a}}i,b^2z) E_{n-k}^{(\alpha -1)}(ay)\nonumber \\&\quad =-2\sum \limits _{l=0,l\ne n}^{n+1}\left({n+1}\right) \frac{B_{n+1-l}}{n+1} b^{n-l} \sum \limits _{k=0}^{l}{}\left( \begin{array}{lll}l\\ k\end{array}\right) a^{n-k}b^{k}\nonumber \\&\qquad \times \sum \limits _{i=0}^{b-1}(-1)^i{}_HE_k^{(\alpha )}(ax+\frac{a}{b}i,a^{2}z) E_{l-k}^{(\alpha -1)}(by) \end{aligned}$$

### Proof

Let

$$\begin{aligned} G(t)=:\frac{2^{2{\alpha }-1}(1-(-e^{bt})^a)e^{ab(x+y)t+a^2b^2zt^2}}{(e^{at}+1)^{\alpha }(e^{bt}+1)^{\alpha }} \end{aligned}$$

In view of definition (1.15), *G*(*t*) has the following expansion:

37 $$\begin{aligned}&G(t)={\left( \frac{2}{e^{bt}+1}\right) ^{\alpha }}e^{abxt+a^2b^2zt^2}{\left( \frac{1-{(-e^{at})}^b}{1+e^{at}}\right) }{\left( \frac{1-{(-e^{bt})}^a}{1-{(-e^{at})}^b} \right) }{\left( \frac{2}{e^{at}+1}\right) ^{{\alpha }-1}}e^{abyt}\nonumber \\&\quad ={\left( -\sum \limits _{n=0}^{\infty }\frac{B_{n}+B_{n}(1)}{n!}abt^{n-1}\right) }{\left( \sum \limits _{i=0}^{b-1}{(-1)^i}\sum \limits _{n=0}^{\infty } {}_HE_n^{(\alpha )}(ax+{\frac{a}{b}}i,a^2z)\frac{(bt)^n}{n!}\right) }\nonumber \\&\qquad \times {\left( \sum \limits _{n=0}^{\infty }E_{n}^{(\alpha -1)}(by)\frac{(bt)^n}{n!}\right) } \end{aligned}$$

Using identity (7) and comparing the coefficients of $\frac{t^n}{n!}$ in the R.H.S. of Eqs. (33) and (37), we get the result (36).

### *Remark 5*

For $z =0$ in Theorem (5), the result reduces to known result of Liu and Wang [2009, Theorem (2.13)].

### **Corollary 5**

*For integers* $n\ge 0,$ $a\ge 1$ *and* $b\ge 1$, *if* *a* *is odd and* *b* *is even, then the following identity holds true:*

38 $$\begin{aligned}&\sum \limits _{k=0}^{n}\left( \begin{array}{lll}n\\ k\end{array}\right) {a^k}{b^{n-k}}\sum \limits _{i=0}^{a-1}{(-1)^i}E_k^{(\alpha )}(bx+{\frac{b}{a}}i) E_{n-k}^{(\alpha -1)}(ay) \nonumber \\&\quad =-2\sum \limits _{l=0,l\ne n}^{n+1}\left( \begin{array}{lll}{n+1}\\ l \end{array}\right) \frac{B_{n+1-l}}{n+1} b^{n-l} \sum \limits _{k=0}^{l}{}\left( \begin{array}{lll}l\\ k\end{array}\right) a^{n-k}b^{k}\nonumber \\&\qquad \times \sum \limits _{i=0}^{b-1}(-1)^iE_k^{(\alpha )}(ax+\frac{a}{b}i) E_{l-k}^{(\alpha -1)}(by) \end{aligned}$$

## Symmetric identities for Hermite–Genocchi polynomials

In this section, we derive some symmetry identities for Hermite–Genocchi polynomials ${}_HG_{n}(x,y)$. We now begin the following theorems.

### **Theorem 6**

*For integers* $n\ge 0$, $a\ge 1$ *and* $b\ge 1$, *if* *a* *and* *b* *have the same parity, then the following identity holds true:*

39 $$\begin{aligned}&\sum \limits _{k=0}^{n}\left( \begin{array}{lll}n\\ k\end{array}\right) {a^k}{b^{n-k+1}}{}_HG_k(bx,b^2y)T_{n-k}(a-1)\nonumber \\&\quad =\sum \limits _{k=0}^{n}\left( \begin{array} {lll}n\\ k\end{array}\right) {b^k}{a^{n-k+1}}{}_HG_k(ax,a^2y)T_{n-k}(b-1) \end{aligned}$$

*and*

40 $$\begin{aligned} b\sum \limits _{i=0}^{a-1}{(-1)^i}a^n{}_HG_n\left(bx+{\frac{b}{a}}i,b^2y\right)=a\sum \limits _{i=0}^{b-1}{(-1)^i}b^n{}_HG_n\left(ax+{\frac{a}{b}}i,a^2y\right) \end{aligned}$$

### Proof

Let

41 $$\begin{aligned} P(t)=: \frac{2abt(1-(-e^{bt})^a)e^{abxt+a^2b^2yt^2}}{(e^{at}+1)(e^{bt}+1)} \end{aligned}$$

We can expand *P*(*t*) as follows

42 $$\begin{aligned}P(t)&=b{\left( \frac{2at}{e^{at}+1}\right) }e^{abxt+a^2b^2yt^2}{\left( \frac{1-(-e^{bt})^a}{e^{bt}+1}\right) }\nonumber \\&=b{\left( \sum \limits _{k=0}^{\infty }{}_HG_{k}(bx,b^2y)\frac{(at)^k}{k!}\right) }{\left( \sum \limits _{n=0}^{\infty }T_{n}(a-1)\frac{(bt)^n}{n!}\right) }\nonumber \\& =b\sum \limits _{n=0}^{\infty }\sum \limits _{k=0}^{\infty }a^kb^n{}_HG_{k}(bx,b^2y)T_{n}(a-1)\frac{t^{n+k}}{n!k!}\nonumber \\P(t)&=\sum \limits _{n=0}^{\infty }{\left[ \sum \limits _{k=0}^{n}\left( \begin{array}{lll}n\\ k\end{array}\right) a^kb^{n-k+1}{}_HG_{k}(bx,b^2y)T_{n-k}(a-1) \right] }\frac{t^n}{n!} \end{aligned}$$

On the other hand

43 $$\begin{aligned} P(t)=\sum \limits _{n=0}^{\infty }{\left[ \sum \limits _{k=0}^{n}\left( \begin{array}{lll}n\\ k\end{array}\right) b^ka^{n-k+1}{}_HG_{k}(ax,a^2y)T_{n-k}(b-1) \right] }\frac{t^n}{n!} \end{aligned}$$

Comparing the coefficients of $\frac{t^n}{n!}$ in the R.H.S. of above Eqs. (42) and (43), yields identity (39).

Now following (41) we expand *P*(*t*) as follows:

44 $$\begin{aligned}P(t)&=b{\left( \frac{2at}{e^{at}+1}\right) }e^{abxt+a^2b^2yt^2}{\left( \frac{1-(-e^{bt})^a}{e^{bt}+1}\right) } \nonumber \\&=b{\left( \frac{2at}{e^{at}+1}\right) }e^{abxt+a^2b^2yt^2}\sum \limits _{i=0}^{a-1}(-1)^ie^{bti} \nonumber \\& =b{\left( \frac{2at}{e^{at}+1}\right) }e^{a^2b^2yt^2}\sum \limits _{i=0}^{a-1}(-1)^ie^{(bx+\frac{b}{a}i)at}\nonumber \\P(t)&=b\sum \limits _{n=0}^{\infty }{\left[ \sum \limits _{i=0}^{a-1}a^n(-1)^i{}_HG_{n}\left(bx+\frac{b}{a}i,b^2y\right)\right] }\frac{t^n}{n!} \end{aligned}$$

On the similar lines, we can show that

45 $$\begin{aligned} P(t)=a\sum \limits _{n=0}^{\infty }{\left[ \sum \limits _{i=0}^{b-1}b^n(-1)^i{}_HG_{n}\left(ax+\frac{a}{b}i,a^2y \right)\right] }\frac{t^n}{n!} \end{aligned}$$

Comparing the coefficients of $\frac{t^n}{n!}$ in the R.H.S. of above Eqs. (44) and (45), yields identity (40).

### *Remark 6*

For $y=0$ in Theorem (6) and (7), the result reduces to known result of Liu and Wang [2009, p. 3358, (4.2) and (4.3)].

### **Corollary 6**

*For integers* $n\ge 0,$ $a\ge 1$ *and* $b\ge 1$, *if* *a* *and* *b* *have the same parity, then the following identity holds true:*

46 $$\begin{aligned}&\sum \limits _{k=0}^{n}\left( \begin{array}{lll}n\\ k\end{array}\right) {a^k}{b^{n-k+1}}G_k(bx)T_{n-k}(a-1)\nonumber \\&\quad =\sum \limits _{k=0}^{n}\left( \begin{array} {lll}n\\ k\end{array}\right) {b^k}{a^{n-k+1}}G_k(ax)T_{n-k}(b-1) \end{aligned}$$

*and*

47 $$\begin{aligned} b\sum \limits _{i=0}^{a-1}{(-1)^i}a^nG_n\left(bx+{\frac{b}{a}}i\right)=a\sum \limits _{i=0}^{b-1}{(-1)^i}b^nG_n\left(ax+{\frac{a}{b}}i\right) \end{aligned}$$

### **Theorem 7**

*For integers* $n\ge 0,$ $a\ge 1$ *and* $b\ge 1$, *if* *a* *is odd and* *b* *is even, then the following identity holds true:*

48 $$\begin{aligned}&\sum \limits _{k=0}^{n}\left( \begin{array}{lll}n\\ k\end{array}\right) a^k b^{n-k+1}{}_HG_k(bx,b^2y)T_{n-k}(a-1)\nonumber \\&\quad =-2\sum \limits _{l=0,l\ne n}^{n+1}\left( \begin{array}{lll}n+1\\ l\end{array}\right) \frac{B_{n+1-l}}{n+1}b^{n-l}\sum \limits _{k=0}^{l} \left( \begin{array}{lll}l\\ k\end{array}\right) b^ka^{n-k+1}{}_HG_{k}(ax,a^2y)T_{l-k}(b-1) \end{aligned}$$

*and*

49 $$\begin{aligned}&b\sum \limits _{i=0}^{a-1}{(-1)^i}a^n{}_HG_n\left(bx+{\frac{b}{a}}i,b^2y\right)\nonumber \\&\quad =-2\sum \limits _{l=0,l\ne n}^{n+1}\left( \begin{array}{lll}n+1\\ l\end{array}\right) \frac{B_{n+1-l}}{n+1}a^{n-l+1}\sum \limits _{i=0}^{b-1}(-1)^ib^n{}_HG_{l}\left(ax+\frac{a}{b}i,a^2y\right) \end{aligned}$$

### Proof

Let

$$\begin{aligned} P(t)=: \frac{2abt(1-(-e^{bt})^a)e^{abxt+a^2b^2yt^2}}{(e^{at}+1)(e^{bt}+1)} \end{aligned}$$

We can expand *P*(*t*) as follows:

50 $$\begin{aligned}&P(t)=a{\left( \frac{2bt}{e^{bt}+1}\right) }e^{abxt+a^2b^2yt^2}{\left( \frac{1-(-e^{bt})^a}{1-(-e^{at})^b}\right) }{\left( \frac{1-(-e^{at})^b}{e^{at}+1}\right) }\nonumber \\&\quad =a{\left( \sum \limits _{k=0}^{\infty }{}_HG_{k}(ax,a^2y)\frac{(bt)^k}{k!}\right) }{\left( -\sum \limits _{n=0}^{\infty }\frac{B_n+B_n(1)}{n!}(abt)^{n-1}\right) } {\left( \sum \limits _{l=0}^{\infty }T_{l}(b-1)\frac{(at)^l}{l!}\right) }\nonumber \\&\quad =-a{\left( \sum \limits _{n=0}^{\infty }\frac{B_n+B_n(1)}{n!}(abt)^{n-1}\right) }{\left( \sum \limits _{l=0}^{\infty }\sum \limits _{k=0}^{l}a^{l-k}b^k{}_HG_{k}(ax,a^2y) T_{l-k}(b-1)\frac{t^l}{{l-k}!k!}\right) }\nonumber \\&P(t)=-a{\left( \sum \limits _{n=0}^{\infty }\frac{B_n+B_n(1)}{n!}(abt)^{n-1}\right) }\sum \limits _{l=0}^{\infty }\sum \limits _{k=0}^{l}\left( \begin{array}{lll}l\\ k \end{array}\right) a^{l-k}{}_HG_{k}(ax,a^2y)T_{l-k}(b-1)\frac{t^{l}}{l!} \end{aligned}$$

Another expansion of *P*(*t*) is

51 $$\begin{aligned}&P(t)=-a{\left( \sum \limits _{n=0}^{\infty }\frac{B_n+B_n(1)}{n!}(abt)^{n-1}\right) }{\left( \frac{2bt}{e^{bt}+1}\right) }e^{abxt+a^2b^2yt^2}{\left( \sum \limits _{i=0} ^{b-1} (-1)^ie^{ati}\right) }\nonumber \\&\quad =-a{\left( \sum \limits _{n=0}^{\infty }\frac{B_n+B_n(1)}{n!}(abt)^{n-1}\right) }{\left( \sum \limits _{i=0}^{b-1}(-1)^i\sum \limits _{k=0}^{\infty }b^k{}_HG_{k} \left(ax+\frac{a}{b}i;a^2y\right)\frac{t^k}{k!}\right) } \end{aligned}$$

Comparing the coefficients of $\frac{t^n}{n!}$ in Eqs. (43) and (50), we obtain the first identity. Also comparing the coefficients of $\frac{t^n}{n!}$ in the R.H.S. of Eqs. (44) and (51), we obtain the second identity.

### *Remark 7*

For $y=0$ in Theorem (7), the result reduces to known result of Liu and Wang [2009, p. 3358, Theorem (4.4)].

### **Corollary 7**

*For integers* $n\ge 0,$ $a\ge 1$ *and* $b\ge 1$, *if* *a* *is odd and* *b* *is even, then the following identity holds true:*

52 $$\begin{aligned}&\sum \limits _{k=0}^{n}\left( \begin{array}{lll}n\\ k\end{array}\right) a^k b^{n-k+1}G_k(bx)T_{n-k}(a-1) \nonumber \\&\quad =-2\sum \limits _{l=0,l\ne n}^{n+1}\left( \begin{array}{lll}n+1\\ l\end{array}\right) \frac{B_{n+1-l}}{n+1}b^{n-l}\sum \limits _{k=0}^{l} \left( \begin{array}{lll}l\\ k\end{array}\right) b^ka^{n-k+1}G_{k}(ax)T_{l-k}(b-1) \end{aligned}$$

*and*

53 $$\begin{aligned}&b\sum \limits _{i=0}^{a-1}{(-1)^i}a^nG_n\left(bx+{\frac{b}{a}}i\right)\nonumber \\&\quad =-2\sum \limits _{l=0,l\ne n}^{n+1}\left( \begin{array}{lll}n+1\\ l\end{array}\right) \frac{B_{n+1-l}}{n+1}a^{n-l+1}\sum \limits _{i=0}^{b-1}(-1)^ib^nG_{l}\left(ax+\frac{a}{b}i \right) \end{aligned}$$

### **Theorem 8**

*For integers* $n\ge 0,$ $a\ge 1$ *and* $b\ge 1$, *if* *a* *is even and* *b* *is odd, then the following identity holds true:*

54 $$\begin{aligned}\sum \limits _{k=0}^{n}\left(\begin{array}{lll}n\\ k\end{array}\right) a^kb^{n-k+1}{}_HG_k(bx,b^2y)T_{n-k}(a-1)&=\sum \limits _{k=0}^{n-1}\left( \begin{array}{lll}n\\ k\end{array}\right) b^ka^{n-k+1}{}_HG_{k}(ax,a^2y)\nonumber \\&\quad\times (E_{n-k}(0)-T_{n-k}(b-1)) \end{aligned}$$

*and*

55 $$\begin{aligned}b\sum \limits _{i=0}^{a-1}(-1)^ia^n{}_HG_{n}\left(bx+\frac{b}{a}i,b^2y\right)&=\sum \limits _{l=0}^{n-1}\left( \begin{array}{lll}n\\ l\end{array}\right) a^{n-l+1}E_{n-l}(0)\nonumber \\&\quad \times \sum \limits _{i=0}^{b-1}(-1)^ib^n{}_HG_l\left(ax+\frac{a}{b}i,a^2y\right) \end{aligned}$$

### Proof

Let

56 $$\begin{aligned} P(t)=:\frac{2abt(1-{(-e^{bt})}^a)}{(e^{at}+1)(e^{bt}+1)}e^{abxt+a^2b^2yt^2} \end{aligned}$$

we expand *P*(*t*) as follows:

57 $$\begin{aligned}P(t)&=a\left( \frac{2bt}{e^{bt}+1}\right) e^{abxt+a^2b^2yt^2}\left( \frac{1-{(-e^{bt})}^a}{1-{(-e^{at})}^b}\right) \left( \frac{(1-{(-e^{at})}^b)}{1+e^{at}} \right) \nonumber \\& =a\left( \sum \limits _{k=0}^{\infty }{}_HG_k(ax,a^2y)\frac{(bt)^k}{k!}\right) \left( \sum \limits _{n=1}^{\infty }E_{n}(0)\frac{(abt)^n}{n!}\right) \left( \sum \limits _{l=0}^{\infty }T_l(b-1)\frac{(at)^l}{l!}\right) \end{aligned}$$

Comparing the coefficients of $\frac{t^n}{n!}$ in the R.H.S. of Eqs. (42) and (57), we have

58 $$\begin{aligned}&\sum \limits _{k=0}^{n}\left( \begin{array}{lll}n\\ k\end{array}\right) {a^k}{b^{n-k+1}}{}_HG_k(bx,b^2y)T_{n-k}(a-1) \nonumber \\&\quad =\sum \limits _{l=0}^{n-1} \left( \begin{array}{lll}n\\ l\end{array}\right) E_{n-l}(0)b^{n-l}\sum \limits _{k=0}^{l}{\left( \begin{array}{lll}l\\ k\end{array}\right) } a^{n-k+1}b^k{}_HG_{k}(ax,a^2y)T_{l-k}(b-1) \nonumber \\&\quad =\sum \limits _{k=0}^{n-1}\left( \begin{array}{lll}n\\ k\end{array}\right) a^{n-k+1}b^k{}_HG_{k}(ax,a^2y)\sum \limits _{l=k}^{n-1} \left( \begin{array}{lll}n-k\\ l-k\end{array}\right) E_{n-l}(0)b^{n-l}T_{l-k}(b-1) \end{aligned}$$

Finally using [Liu and Wang (2009), p. 3348, (2.4)], we arrive at the desired result (54).

Now following (56), we expand *P*(*t*) as follows:

59 $$\begin{aligned}P(t) &=a\left( \frac{2bt}{e^{bt}+1}\right) e^{abxt+a^2b^2yt^2}\left( \frac{1-{(-e^{bt})}^a}{1-{(-e^{at})}^b}\right) \left( \frac{(1-{(-e^{at})}^b)}{1+e^{at}} \right) \nonumber \\ \quad &=a{\left( \sum \limits _{n=1}^{\infty }E_{n}(0)\frac{(abt)^n}{n!}\right) }{\left( \sum \limits _{l=0}^{\infty }\sum \limits _{i=0}^{b-1}(-1)^ib^l{}_HG_{l}\left(ax+\frac{a}{b}i, a^2y\right)\frac{t^l}{l!}\right) } \end{aligned}$$

which on equating the coefficients of $\frac{t^n}{n!}$ in the R.H.S. of Eqs. (44) and (59), gives the result (55).

### *Remark 8*

For $y=0$ in Theorem (8), the result reduces to known result of Liu and Wang [2009, p. 3359, Theorem (4.6)].

### **Corollary 8**

*For integers* $n\ge 0$ , $a\ge 1$ *and* $b\ge 1$, *if* *a* *is even and* *b* *is odd, then the following identity holds true:*

60 $$\begin{aligned}\sum \limits _{k=0}^{n}\left( \begin{array}{lll}n\\ k\end{array}\right) a^kb^{n-k+1}G_k(bx)T_{n-k}(a-1)&=\sum \limits _{k=0}^{n-1}\left( \begin{array}{lll}n\\ k\end{array}\right) b^ka^{n-k+1}G_{k}(ax) \nonumber \\&\quad \times (E_{n-k}(0)-T_{n-k}(b-1)) \end{aligned}$$

*and*

61 $$\begin{aligned}b\sum \limits _{i=0}^{a-1}(-1)^ia^nG_{n}\left(bx+\frac{b}{a}i\right)&=\sum \limits _{l=0}^{n-1}\left( \begin{array}{lll}n\\ l\end{array}\right) a^{n-l+1}E_{n-l}(0)\nonumber \\&\quad \times \sum \limits _{i=0}^{b-1}(-1)^ib^nG_l\left(ax+\frac{a}{b}i\right) \end{aligned}$$

## Mixed type identities

In this section, we establish some mixed type identities involving the generalized Hermite–Bernoulli and Euler polynomials of order $\alpha$. Throughout this section $\alpha$ will be taken as an arbitrary real or complex parameter.

### **Theorem 9**

*For integers* $n\ge 1$, $a\ge 1$ *and* $b\ge 1$, *if a is even, then the following identity holds true:*

62 $$\begin{aligned}&\sum \limits _{k=0}^{n}\left( \begin{array}{lll}n\\ k\end{array}\right) {b^k}{a^{n-k}}{}_HB_{n-k}^{(\alpha )}(bx,b^{2}z)\sum \limits _{i=0}^{k}\left( \begin{array} {lll}k\\ i\end{array}\right) T_i(a-1)E_{k-i}^{(\alpha -1)}(ay) \nonumber \\&\quad =-\frac{n}{2}\sum \limits _{k=0}^{n-1}\left( \begin{array}{lll}{n-1}\\ k\end{array}\right) {a^{k+1}} {b^{n-k-1}}{}_HE_{n-1-k}^{(\alpha )}(ax,a^{2}z)\nonumber \\&\qquad \times \sum \limits _{i=0}^{k}\left( \begin{array}{lll}k\\ i\end{array}\right) S_i(b-1)B_{k-i}^{(\alpha -1)}(by) \end{aligned}$$

*and*

63 $$\begin{aligned}&\sum \limits _{k=0}^{n}\left( \begin{array}{lll}n\\ k\end{array}\right) {a^k}{b^{n-k}}\sum \limits _{i=0}^{a-1}(-1)^i{}_HB_{k}^{(\alpha )}\left(bx+\frac{b}{a}i,b^{2}z\right) E_{n-k}^{(\alpha -1)}(ay) \nonumber \\&\quad =-\frac{n}{2}\sum \limits _{k=0}^{n-1}\left( \begin{array}{lll}{n-1}\\ k\end{array}\right) {b^k}{a^{n-k}}\sum \limits _{i=0}^{b-1} {}_HE_{k}^{(\alpha )}\left(ax+\frac{a}{b}i,a^{2}z\right)B_{n-k-1}^{(\alpha -1)}(by) \end{aligned}$$

### Proof

Let

64 $$\begin{aligned}H(t)&=:\frac{2^{\alpha -1}a^{\alpha }t^{\alpha }(1-(-e^{bt})^a)e^{ab(x+y)t+a^2b^2zt^2}}{(e^{at}-1)^{\alpha }(e^{bt}+1)^{\alpha }}\\ & ={\left( \frac{at}{e^{at}-1}\right) ^{\alpha }}e^{abxt+a^2b^2zt^2}{\left( \frac{1-{(-e^{bt})}^a}{1+e^{bt}}\right) }{\left( \frac{2}{e^{bt}+1}\right) ^{{\alpha }-1}}e^{abyt}\nonumber \\& ={\left( \sum \limits _{n=0}^{\infty }{}_HB_{n}^{(\alpha )}(bx,b^2z)\frac{(at)^n}{n!}\right) }{\left( \sum \limits _{i=0}^{\infty } T_i(a-1)\frac{(bt)^i}{i!}\right) }{\left( \sum \limits _{k=0}^{\infty }E_k^{(\alpha -1)}(ay)\frac{(bt)^k}{k!}\right) }\nonumber \\&\end{aligned}$$

65 $$\begin{aligned}H(t)={\left( \sum \limits _{n=0}^{\infty }{}_HB_{n}^{(\alpha )}(bx,b^2z)\frac{(at)^n}{n!}\right) }{\left( \sum \limits _{k=0}^{\infty } \sum \limits _{i=0}^{k}\left( \begin{array}{lll}k\\ i\end{array}\right) b^kT_i(a-1)E_{k-i}^{(\alpha -1)}(ay)\frac{t^k}{k!}\right) } \end{aligned}$$

Since *a* is even, we also have

66 $$\begin{aligned}H(t)&=-\frac{ta}{2}{\left( \frac{2}{e^{bt}+1}\right) ^{\alpha }}e^{abxt+a^2b^2zt^2}{\left( \frac{e^{abt}-1}{e^{at}-1}\right) }{\left( \frac{at}{e^{at}-1}\right) ^{{\alpha }-1}}e^{abyt}\nonumber \\&=-\frac{ta}{2}{\left( \sum \limits _{n=0}^{\infty }{}_HE_{n}^{(\alpha )}(ax,a^2z)\frac{(bt)^n}{n!}\right) }{\left( \sum \limits _{i=0}^{\infty } S_i(b-1)\frac{(at)^i}{i!}\right) }{\left( \sum \limits _{k=0}^{\infty }B_k^{(\alpha -1)}(by)\frac{(at)^k}{k!}\right) }\nonumber \\&=-\frac{ta}{2}{\left( \sum \limits _{n=0}^{\infty }{}_HE_{n}^{(\alpha )}(ax,a^2z)\frac{(bt)^n}{n!}\right) }{\left( \sum \limits _{k=0}^{\infty } \sum \limits _{i=0}^{k}\left( \begin{array}{lll}k\\ i\end{array}\right) a^kS_i(b-1)B_{k-i}^{(\alpha -1)}(by)\frac{t^k}{k!}\right) } \end{aligned}$$

Comparing coefficients of $\frac{t^n}{n!}$ in the R.H.S. of above Eqs. (65) and (66), yields the first identity (62).

Again, we expand *H*(*t*) as follows:

67 $$\begin{aligned}H(t)&={\left( \frac{at}{e^{at}-1}\right) ^{\alpha }}e^{abxt+a^2b^2zt^2}{\left( \frac{1-{(-e^{bt})}^a}{1+e^{bt}}\right) }{\left( \frac{2}{e^{bt}+1}\right) ^{{\alpha }-1}}e^{abyt}\nonumber \\& ={\left( \frac{at}{e^{at}-1}\right) ^{\alpha }}e^{abxt+a^2b^2zt^2}{\left( \sum \limits _{i=0}^{a-1}(-1)^ie^{bti}\right) }{\left( \frac{2}{e^{bt}+1}\right) ^{{\alpha }-1}}e^{abyt}\nonumber \\&={\left( \frac{at}{e^{at}-1}\right) ^{\alpha }}e^{a^2b^2zt^2}{\left( \sum \limits _{i=0}^{a-1}(-1)^ie^{(bx+\frac{b}{a}i)}at \right) }{\left( \sum \limits _{n=0}^{\infty }E_{n}^{(\alpha -1)}(ay)\frac{(bt)^n}{n!}\right) }\nonumber \\H(t)&={\left( \sum \limits _{i=0}^{a-1}(-1)^i\sum \limits _{k=0}^{\infty }{}_HB_{k}^{(\alpha )}({bx+\frac{b}{a}i;b^2z})\frac{(at)^k}{k!}\right) }{\left( \sum \limits _{n=0}^{\infty }E_{n}^{(\alpha -1)}(ay)\frac{(bt)^n}{n!}\right) } \end{aligned}$$

On similar lines, we can expand *H*(*t*) as follows:

68 $$\begin{aligned}H(t)&=-\frac{ta}{2}{\left( \frac{2}{e^{bt}+1}\right) ^{\alpha }}e^{abxt+a^2b^2zt^2}{\left( \frac{e^{abt}-1}{e^{at}-1}\right) }{\left( \frac{at}{e^{at}-1}\right) ^{{\alpha }-1}}e^{abyt}\nonumber \\& =-\frac{ta}{2}{\left( \frac{2}{e^{bt}+1}\right) }^{\alpha }e^{abxt+a^2b^2zt^2}{\left( \sum \limits _{i=0}^{b-1}e^{ati}\right) }{\left( \frac{at}{e^{at}-1} \right) }^{{\alpha }-1}e^{abyt}\nonumber \\H(t)&=-\frac{ta}{2}{\left( \sum \limits _{i=0}^{b-1}\sum \limits _{k=0}^{\infty }{}_HE_{k}^{(\alpha )}\left(ax+\frac{a}{b}i,a^{2}z\right)\frac{(bt)^k}{k!}\right) } {\left( \sum \limits _{n=0}^{\infty }B_{n}^{(\alpha -1)}(by)\frac{(at)^n}{n!}\right) } \end{aligned}$$

Comparing the coefficients of $\frac{t^n}{n!}$ in the R.H.S of above Eqs. (67) and (68), we get the result (63).

### *Remark 9*

For $z=0$ in Theorem (9), the result reduces to known result of Liu and Wang [2009, p. 3353, Theorem (3.1)].

### **Corollary 9**

*For integers* $n\ge 1$, $a\ge 1$ *and* $b\ge 1$, *if a is even, then the following identity holds true:*

69 $$\begin{aligned}&\sum \limits _{k=0}^{n}\left( \begin{array}{lll}n\\ k\end{array}\right) {b^k}{a^{n-k}}B_{n-k}^{(\alpha )}(bx)\sum \limits _{i=0}^{k}\left( \begin{array} {lll}k\\ i\end{array}\right) T_i(a-1)E_{k-i}^{(\alpha -1)}(ay)\nonumber \\&\quad =-\frac{n}{2}\sum \limits _{k=0}^{n-1}\left( \begin{array}{lll}{n-1}\\ k\end{array}\right) {a^{k+1}} {b^{n-k-1}}E_{n-1-k}^{(\alpha )}(ax)\nonumber \\&\qquad \times \sum \limits _{i=0}^{k}\left( \begin{array}{lll}k\\ i\end{array}\right) S_i(b-1)B_{k-i}^{(\alpha -1)}(by) \end{aligned}$$

*and*

70 $$\begin{aligned}&\sum \limits _{k=0}^{n}\left( \begin{array}{lll}n\\ k\end{array}\right) {a^k}{b^{n-k}}\sum \limits _{i=0}^{a-1}(-1)^iB_{k}^{(\alpha )}\left(bx+\frac{b}{a}i\right) E_{n-k}^{(\alpha -1)}(ay) \nonumber \\&\quad =-\frac{n}{2}\sum \limits _{k=0}^{n-1}\left( \begin{array}{lll}{n-1}\\ k\end{array}\right) {b^k}{a^{n-k}}\sum \limits _{i=0}^{b-1} E_{k}^{(\alpha )}\left(ax+\frac{a}{b}i\right)B_{n-k-1}^{(\alpha -1)}(by) \end{aligned}$$

### **Theorem 10**

*For integers* $n\ge 1$, $a\ge 1$ *and* $b\ge 1$, *if* *a* *is odd, then the following identity holds true:*

71 $$\begin{aligned}&\sum \limits _{k=0}^{n}\left( \begin{array}{lll}n\\ k\end{array}\right) {b^k}{a^{n-k}}{}_HB_{n-k}^{(\alpha )}(bx,b^{2}z)\sum \limits _{i=0}^{k}\left( \begin{array} {lll}k\\ i\end{array}\right) T_i(a-1)E_{k-i}^{(\alpha -1)}(ay) \nonumber \\&\quad =\sum \limits _{l=0,l\ne n-1}^{n}\left( \begin{array}{lll}n\\ l\end{array}\right) B_{n-l}{a^{n-l}}\sum \limits _{k=0,}^{l}\left( \begin{array}{lll}l\\ k\end{array}\right) {b^{n-k-1}}a^k{}_HE_{l-k}^{(\alpha )}(ax,a^{2}z)\nonumber \\&\qquad \times \sum \limits _{i=0}^{k}\left( \begin{array}{lll}k\\ i\end{array}\right) S_i(b-1)B_{k-i}^{(\alpha -1)}(by) \end{aligned}$$

*and*

72 $$\begin{aligned}&\sum \limits _{k=0}^{n}\left( \begin{array}{lll}n\\ k\end{array}\right) {a^k}{b^{n-k}}\sum \limits _{i=0}^{a-1}(-1)^i{}_HB_{k}^{(\alpha )}\left(bx+\frac{b}{a}i,b^{2}z\right) E_{n-k}^{(\alpha -1)}(ay)\nonumber \\&\quad =\sum \limits _{l=0,l\ne n-1}^{n}\left( \begin{array}{lll}n\\ l\end{array}\right) B_{n-l}{b^{n-l-1}}\sum \limits _{k=0}^{l} \left( \begin{array}{lll}l\\ k\end{array}\right) {b^k}{a^{n-k}}\nonumber \\&\qquad \times \sum \limits _{i=0}^{b-1}{}_HE_{k}^{(\alpha )}\left(ax+\frac{a}{b}i,a^{2}z\right)B_{l-k}^{(\alpha -1)}(by) \end{aligned}$$

### Proof

Let

$$\begin{aligned} H(t)=:\frac{t^{\alpha }(1-(-e^{bt})^a)e^{ab(x+y)t+a^2b^2zt^2}}{(e^{at}-1)^{\alpha }(e^{bt}+1)^{\alpha }} \end{aligned}$$

We expand *H*(*t*) as follows:

73 $$\begin{aligned}H(t)&=\frac{-t}{2^{\alpha }{a^{\alpha -1}}}{\left( \frac{2}{e^{bt}+1}\right) ^{\alpha }}e^{abxt+a^2b^2zt^2}{\left( \frac{1-{(-e^{bt})}^a}{1-{(-e^{at})}^b}\right) } {\left( \frac{e^{abt}-1}{e^{at}-1}\right) }\nonumber \\&\quad \times {\left( \frac{at}{e^{at}-1}\right) ^{{\alpha }-1}}e^{abyt} \nonumber \\& =\frac{t}{2^{\alpha }{a^{\alpha -1}}}{\left( \sum \limits _{l=0}^{\infty }{}_HE_{l}^{(\alpha )}(ax,a^{2}z)\frac{(bt)^l}{l!}\right) }{\left( \sum \limits _{n=0}^ {\infty }\frac{B_n+B_n(1)}{n!}{(abt)}^{n-1}\right) }\nonumber \\&\quad \times {\left( \sum \limits _{i=0}^{\infty }S_i(b-1)\frac{(at)^i}{i!}\right) }{\left( \sum \limits _{k=0}^{\infty } B_{k}^{(\alpha -1)}(by)\frac{(at)^k}{k!}\right) } \end{aligned}$$

Equating the coefficients of $\frac{t^n}{n!}$ in the R.H.S. of above Eqs. (65) and (73) and using the identity (7), we obtain the first identity (71).

On the other hand

74 $$\begin{aligned}H(t)&=\frac{-t}{2^{\alpha }{a^{\alpha -1}}}{\left( \frac{2}{e^{bt}+1}\right) ^{\alpha }}e^{abxt+a^2b^2zt^2}{\left( \frac{1-{(-e^{bt})}^a}{1-{(-e^{at})}^b}\right) } {\left( \frac{e^{abt}-1}{e^{at}-1}\right) }{\left( \frac{at}{e^{at}-1}\right) ^{\alpha -1}}e^{abyt}\nonumber \\ \quad &=\frac{-t}{2^{\alpha }{a^{\alpha -1}}}{\left( -\sum \limits _{n=0}^{\infty }\frac{B_n+B_n(1)}{n!}{abt}^{n-1}\right) }{\left( \frac{2}{e^{bt}+1}\right) ^{\alpha }} e^{abxt+a^2b^2zt^2}\nonumber \\ &\quad \times {\left( \sum \limits _{i=0}^{b-1}e^{ati}\right) }{\left( \sum \limits _{l=0}^{\infty }B_{l}^{(\alpha -1)}(by)\frac{(at)^l}{l!}\right) }\nonumber \\ H(t)&=\frac{t}{2^{\alpha }{a^{\alpha -1}}}{\left( \sum \limits _{n=0}^{\infty }\frac{B_n+B_n(1)}{n!}{abt}^{n-1}\right) }{\left( \sum \limits _{i=0}^{b-1}\sum \limits _{k=0} ^{\infty }{}_HE_{k}^{(\alpha )}(ax+\frac{a}{b}i,a^{2}z)\frac{(bt)^k}{k!}\right) }\nonumber \\&\quad \times {\left( \sum \limits _{l=0}^{\infty }B_{l}^{(\alpha -1)}(by)\frac{(at)^l}{l!}\right) } \end{aligned}$$

Equating the coefficients of $\frac{t^n}{n!}$ in the R.H.S. of above Eqs. (67) and (74), yields the result (72).

### *Remark 10*

For $z=0$ in Theorem (10), the result reduces to known result of Liu and Wang [2009, p. 3354, Theorem (3.6)].

### **Corollary 10**

*For integers* $n\ge 1$, $a\ge 1$ *and* $b\ge 1$, *if* *a* *is odd, then the following identity holds true:*

75 $$\begin{aligned}&\sum \limits _{k=0}^{n}\left( \begin{array}{lll}n\\ k\end{array}\right) {b^k}{a^{n-k}}B_{n-k}^{(\alpha )}(bx)\sum \limits _{i=0}^{k}\left( \begin{array} {lll}k\\ i\end{array}\right) T_i(a-1)E_{k-i}^{(\alpha -1)}(ay)\nonumber \\&\quad =\sum \limits _{l=0,l\ne n-1}^{n}\left( \begin{array}{lll}n\\ l\end{array}\right) B_{n-l}{a^{n-l}}\sum \limits _{k=0,}^{l}\left( \begin{array}{lll}l\\ k\end{array}\right) {b^{n-k-1}}a^kE_{l-k}^{(\alpha )}(ax) \nonumber \\&\qquad \times \sum \limits _{i=0}^{k}\left( \begin{array}{lll}k\\ i\end{array}\right) S_i(b-1)B_{k-i}^{(\alpha -1)}(by) \end{aligned}$$

*and*

76 $$\begin{aligned}&\sum \limits _{k=0}^{n}\left( \begin{array}{lll}n\\ k\end{array}\right) {a^k}{b^{n-k}}\sum \limits _{i=0}^{a-1}(-1)^iB_{k}^{(\alpha )}\left(bx+\frac{b}{a}i\right) E_{n-k}^{(\alpha -1)}(ay)\nonumber \\&\quad =\sum \limits _{l=0,l\ne n-1}^{n}\left( \begin{array}{lll}n\\ l\end{array}\right) B_{n-l}{b^{n-l-1}}\sum \limits _{k=0}^{l} \left( \begin{array}{lll}l\\ k\end{array}\right) {b^k}{a^{n-k}} \nonumber \\&\qquad \times \sum \limits _{i=0}^{b-1}E_{k}^{(\alpha )}\left(ax+\frac{a}{b}i\right)B_{l-k}^{(\alpha -1)}(by) \end{aligned}$$

### **Theorem 11**

*For integers* $n\ge 1$, $a\ge 1$ *and* $b\ge 1$ , *the following identity holds true:*

77 $$\begin{aligned}&\sum \limits _{k=0}^{n}\left( \begin{array}{lll}n\\ k\end{array}\right) {b^k}{a^{n-k}}{}_HB_{n-k}^{(\alpha )}(bx,b^{2}z)\sum \limits _{i=0}^{k}\left( \begin{array} {lll}k\\ i\end{array}\right) T_i(a-1)E_{k-i}^{(\alpha -1)}(ay)\nonumber \\&\quad =\sum \limits _{k=0}^{n}\left( \begin{array}{lll}n\\ k\end{array}\right) {a^k}{b^{n-k}}\sum \limits _{i=0} ^{a-1}(-1)^i{}_HB_{k}^{(\alpha )}\left(bx+\frac{b}{a}i,b^{2}z\right)E_{n-k}^{(\alpha -1)}(ay) \end{aligned}$$

### Proof

Let

78 $$\begin{aligned}&H(t)=:\frac{2^{\alpha -1}a^{\alpha }t^{\alpha }(1-(-e^{bt})^a)e^{ab(x+y)t+a^2b^2zt^2}}{(e^{at}-1)^{\alpha }(e^{bt}+1)^{\alpha }} \nonumber \\&\quad ={\left( \sum \limits _{n=0}^{\infty }{}_HB_{n}^{(\alpha )}(bx,b^2z)\frac{(at)^n}{n!}\right) }{\left( \sum \limits _{i=0}^{\infty } T_i(a-1)\frac{(bt)^i}{i!}\right) }{\left( \sum \limits _{k=0}^{\infty }E_k^{(\alpha -1)}(ay)\frac{(bt)^k}{k!}\right) }\nonumber \\&\quad ={\left( \sum \limits _{n=0}^{\infty }{}_HB_{n}^{(\alpha )}(bx,b^2z)\frac{(at)^n}{n!}\right) }{\left( \sum \limits _{k=0}^{\infty } \sum \limits _{i=0}^{k}\left( \begin{array}{lll}k\\ i\end{array}\right) b^kT_i(a-1)E_{k-i}^{(\alpha -1)}(ay)\frac{t^k}{k!}\right) } \end{aligned}$$

On the other hand

79 $$\begin{aligned}H(t)&={\left( \frac{at}{e^{at}-1}\right) ^{\alpha }}e^{abxt+a^2b^2zt^2}{\left( \frac{1-{(-e^{bt})}^a}{1+e^{bt}}\right) }{\left( \frac{2}{e^{bt}+1}\right) ^{{\alpha }-1}} e^{abyt}\nonumber \\& ={\left( \frac{at}{e^{at}-1}\right) ^{\alpha }}e^{abxt+a^2b^2zt^2}{\left( \sum \limits _{i=0}^{a-1}(-1)^ie^{bti}\right) }{\left( \frac{2}{e^{bt}+1}\right) ^{{\alpha }-1}} e^{abyt}\nonumber \\& ={\left( \frac{at}{e^{at}-1}\right) ^{\alpha }}e^{a^2b^2zt^2}{\left( \sum \limits _{i=0}^{a-1}(-1)^ie^{(bx+\frac{b}{a}i)}at \right) }{\left( \sum \limits _{n=0}^{\infty }E_{n}^{(\alpha -1)}(ay)\frac{(bt)^n}{n!}\right) } \end{aligned}$$

Comparing the coefficients of $\frac{t^n}{n!}$ in the R.H.S of above Eqs. (78) and (79), yields the desired result.

### *Remark 11*

For $z=0$ in Theorem (11), the result reduces to known result of Liu and Wang [2009, p. 3356, Theorem (3.9)].

### **Corollary 11**

*For integers* $n\ge 1$, $a\ge 1$ *and* $b\ge 1$, *the following identity holds true:*

80 $$\begin{aligned}&\sum \limits _{k=0}^{n}\left( \begin{array}{lll}n\\ k\end{array}\right) {b^k}{a^{n-k}}B_{n-k}^{(\alpha )}(bx)\sum \limits _{i=0}^{k}\left( \begin{array} {lll}k\\ i\end{array}\right) T_i(a-1)E_{k-i}^{(\alpha -1)}(ay)\nonumber \\&\quad =\sum \limits _{k=0}^{n}\left( \begin{array}{lll}n\\ k\end{array}\right) {a^k}{b^{n-k}}\sum \limits _{i=0} ^{a-1}(-1)^iB_{k}^{(\alpha )}\left(bx+\frac{b}{a}i\right)E_{n-k}^{(\alpha -1)}(ay) \end{aligned}$$

### **Theorem 12**

*For integers* $n\ge 1$, $a\ge 1$ *and* $b\ge 1$, *the following identity holds true:*

81 $$\begin{aligned}&\sum \limits _{k=0}^{n}\left( \begin{array}{lll}n\\ k\end{array}\right) {b^k}{a^{n-k}}{}_HE_{n-k}^{(\alpha )}(bx,b^{2}z)\sum \limits _{i=0}^{k}\left( \begin{array} {lll}k\\ i\end{array}\right) S_i(a-1)B_{k-i}^{(\alpha -1)}(ay)\nonumber \\&\quad =\sum \limits _{k=0}^{n}\left( \begin{array}{lll}n\\ k\end{array}\right) {a^k}{b^{n-k}}\sum \limits _{i=0} ^{a-1}{}_HE_{k}^{(\alpha )}\left(bx+\frac{b}{a}i,b^{2}z\right)B_{n-k}^{(\alpha -1)}(ay) \end{aligned}$$

### Proof

Let

82 $$\begin{aligned}H(t)&=:\frac{t^{\alpha }(1-(-e^{bt})^a)e^{ab(x+y)t+a^2b^2zt^2}}{(e^{at}+1)^{\alpha }(e^{bt}-1)^{\alpha }}\nonumber \\H(t)&=\frac{-t}{2^{\alpha }{b^{\alpha -1}}}{\left( \frac{2}{e^{at}+1}\right) ^{{\alpha }}}e^{abxt+a^2b^2zt^2}{\left( \frac{e^{abt}-1}{e^{bt}-1}\right) } {\left( \frac{bt}{e^{bt}-1}\right) ^{{\alpha }-1}}e^{abyt}\nonumber \\& =\frac{-t}{2^{\alpha }{b^{\alpha -1}}}{\left( \sum \limits _{n=0}^{\infty }{}_HE_{n}^{(\alpha )}(bx,b^{2}z)\frac{(at)^n}{n!}\right) }{\left( \sum \limits _{i=0} ^{\infty }S_i(a-1)\frac{(bt)^i}{i!}\right) }{\left( \sum \limits _{k=0}^{\infty }B_{k}^{(\alpha -1)}(ay)\frac{(bt)^k}{k!}\right) }\nonumber \\& =\frac{-t}{2^{\alpha }{b^{\alpha -1}}}{\left( \sum \limits _{n=0}^{\infty }{}_HE_{n}^{(\alpha )}(bx,b^{2}z)\frac{(at)^n}{n!}\right) } {\left( \sum \limits _{k=0}^{\infty }\sum \limits _{i=0}^{k}b^kS_i(a-1)B_{k-i}^{(\alpha -1)}(ay)\frac{t^k}{{(k-i)!}i!}\right) } \end{aligned}$$

Another expansion of *H*(*t*) is as follows:

83 $$\begin{aligned}H(t)&=\frac{-t}{2^{\alpha }{b^{\alpha -1}}}{\left( \frac{2}{e^{at}+1}\right) ^{{\alpha }}}e^{abxt+a^2b^2zt^2}{\left( \frac{e^{abt}-1}{e^{bt}-1}\right) } {\left( \frac{bt}{e^{bt}-1}\right) ^{{\alpha }-1}}e^{abyt}\nonumber \\& =\frac{-t}{2^{\alpha }{b^{\alpha -1}}}{\left( \frac{2}{e^{at}+1}\right) ^{{\alpha }}}e^{abxt+a^2b^2zt^2}{\left( \sum \limits _{i=0}^{a-1}e^{bti}\right) } {\left( \sum \limits _{n=0}^{\infty }B_{n}^{(\alpha -1)}(ay)\frac{(bt)^n}{n!}\right) }\nonumber \\& =\frac{-t}{2^{\alpha }{b^{\alpha -1}}}{\left( \sum \limits _{n=0}^{\infty }B_{n}^{(\alpha -1)}(ay)\frac{(bt)^n}{n!}\right) }{\left( \sum \limits _{i=0}^{a-1} \sum \limits _{k=0}^{\infty }{}_HE_{k}^{(\alpha )}\left(bx+\frac{b}{a}i,b^2z\right)\frac{(at)^k}{k!}\right) } \end{aligned}$$

Equating the coefficients of $\frac{t^n}{n!}$ in the R.H.S. of above Eqs. (82) and (83), yields the desired result.

### *Remark 12*

For $z=0$ in Theorem (12), the result reduces to known result of Liu and Wang [2009, p. 3356, Theorem (3.11)].

### **Corollary 12**

*For integers* $n\ge 1$, $a\ge 1$ *and* $b\ge 1$, *the following identity holds true:*

84 $$\begin{aligned}&\sum \limits _{k=0}^{n}\left( \begin{array}{lll}n\\ k\end{array}\right) {b^k}{a^{n-k}}E_{n-k}^{(\alpha )}(bx)\sum \limits _{i=0}^{k}\left( \begin{array} {lll}k\\ i\end{array}\right) S_i(a-1)B_{k-i}^{(\alpha -1)}(ay)\nonumber \\&\quad =\sum \limits _{k=0}^{n}\left( \begin{array}{lll}n\\ k\end{array}\right) {a^k}{b^{n-k}}\sum \limits _{i=0} ^{a-1}E_{k}^{(\alpha )}(bx+\frac{b}{a}i)B_{n-k}^{(\alpha -1)}(ay) \end{aligned}$$

## Conclusion

The definition and generating function of the generalized Hermite–Bernoulli, Euler and Hermite–Genocchi polynomials plays a major role in obtaining new expansions, identities and representations. We can introduce and study a class of related generalized polynomials by defining Gould-Hopper Bernoulli, Euler and Genocchi polynomials.

85 $$\begin{aligned} \left( \frac{t}{e^t-1}\right) ^{\alpha }e^{xt+yt^r}=\sum \limits _{n=0}^{\infty }{}_HB_n^{(\alpha , r)}(x,y)\frac{t^n}{n!} \end{aligned}$$

The Eq. (18) may be derived from (85) for $r = 2$.

This process can easily be extended to establish multi-variable Hermite–Bernoulli, Euler and Genocchi polynomials and Hermite Apostle type Bernoulli, Euler and Genocchi polynomials.
